# Voluntary self-initiation of the stimuli onset improves working memory and accelerates visual and attentional processing

**DOI:** 10.1016/j.heliyon.2022.e12215

**Published:** 2022-12-10

**Authors:** Rocio Loyola-Navarro, Cristóbal Moënne-Loccoz, Rodrigo C. Vergara, Alexandre Hyafil, Francisco Aboitiz, Pedro E. Maldonado

**Affiliations:** a Departamento de Neurociencia, Universidad de Chile, Santiago, Chile; b Biomedical Neuroscience Institute (BNI), Santiago, Chile; c Departamento de Educación Diferencial, Universidad Metropolitana de Ciencias de la Educación, Santiago, Chile; d Center for Advanced Research in Education, Institute of Education, Universidad de Chile, Santiago, Chile; e Departamento de Ciencias de la Salud, Pontificia Universidad Católica de Chile, Santiago, Chile; f Departamento de Kinesiología, Universidad Metropolitana de Ciencias de la Educación, Santiago, Chile; g Centre de Recerca Matemàtica, Barcelona, Spain; h Escuela de Medicina, Pontificia Universidad Católica de Chile, Santiago, Chile; i Centro Nacional de Inteligencia Artificial (CENIA), Santiago, Chile; j Centro de Investigación en Educación, Universidad Metropolitana de Ciencias de la Educación (CIE-UMCE), Santiago, Chile

**Keywords:** EEG, Enaction, Embodied cognition, ERP, Sternberg task, Temporal expectation

## Abstract

The ability of an organism to voluntarily control the stimuli onset modulates perceptual and attentional functions. Since stimulus encoding is an essential component of working memory (WM), we conjectured that controlling the initiation of the perceptual process would positively modulate WM. To corroborate this proposition, we tested twenty-five healthy subjects in a modified-Sternberg WM task under three stimuli presentation conditions: an automatic presentation of the stimuli, a self-initiated presentation of the stimuli (through a button press), and a self-initiated presentation with random-delay stimuli onset. Concurrently, we recorded the subjects' electroencephalographic signals during WM encoding. We found that the self-initiated condition was associated with better WM accuracy, and earlier latencies of N1, P2 and P3 evoked potential components representing visual, attentional and mental review of the stimuli processes, respectively. Our work demonstrates that self-initiated stimuli enhance WM performance and accelerate early visual and attentional processes deployed during WM encoding. We also found that self-initiated stimuli correlate with an increased attentional state compared to the other two conditions, suggesting a role for temporal stimuli predictability. Our study remarks on the relevance of self-control of the stimuli onset in sensory, attentional and memory updating processing for WM.

## 1 Introduction

Classic Working Memory (WM) paradigms require subjects to passively attend to task-relevant stimuli presented without control of the participants. In contrast, in everyday situations, relevant stimuli to WM tasks may appear to our sensory system due to the agent's active control, such as what occurs when we turn the page of a book or slide the mobile screen to browse a web page. When the agent initiates the presentation of the stimuli via a motor action (hereafter, self-initiation of the stimuli), behavior and its underlying neural mechanisms are modulated in several cognitive processes such as perception and attention (Kumar et al., 2015; Nittono et al., 2003; Nittono, 2004, 2006; Waszak and Herwig, 2007). This idea seems to be in line with the embodied cognition perspective (O'Regan & Noë, 2001; Varela et al., 2016), which states that subjects' bodies, particularly their motor systems, influence cognition. Nevertheless, it remains unknown whether the self-initiation of the stimuli modulates WM. Additionally, it has not been established if this influence is achieved by modulating sensory, attentional processes or later updating mechanisms of stimuli in memory. Revealing if self-initiated stimuli modulate WM improves our understanding of the WM mechanism in closer to real-life situations, hallmarking participants' role as agents of their cognitive process.

What are the mechanisms deployed during the self-initiation of the stimuli that can modulate WM encoding? Evidence in perceptual and attentional tasks has shown that self-initiated stimuli modulate both sensory and attentional neural processing (Concha-Miranda et al., 2019; Nittono et al., 2003; Walsh and Haggard, 2008; Stenner et al., 2015). This finding is relevant in WM's context since WM has been proved to rely on both perceptual (Teng and Kravitz, 2019; Zhang et al., 2019) and attentional processing (Postle, 2006). Furthermore, attention is proposed as a critical component of WM, being responsible for maintaining items and for the WM span limits (Cowan, 2005).

How does self-initiation of the stimuli modulate perception? One proposed mechanism is the modulation that motor activity exerts on early sensory cortices (Concha-Miranda et al., 2019; Schroeder et al., 2010; Stenner et al., 2015). It has been widely shown that self-initiated stimuli (controlled by a button press) attenuate sensory response in the auditory (Knolle et al., 2013; SanMiguel et al., 2013; Sowman et al., 2012) and somatosensory domains (Blakemore et al., 1999; Hesse et al., 2010). In the visual domain, however, evidence is heterogenous, with some studies showing visual sensory attenuation (Mifsud et al., 2018), and others showing that self-initiated stimulus does not induce visual cortex’ activity attenuation, but attenuated later non-domain specific processing (Hughes and Waszak, 2011; Schafer and Marcus, 1973). Eye movements such as saccades can also control the stimuli onset in a self-initiated way. During saccades the visual sensitivity is first reduced (Diamond et al., 2000), followed by a firing rate increase (McFarland et al., 2015). Moreover, local field potential amplitude is larger when locked to a saccade than when locked to fixation (Ito et al., 2011). In humans, a similar modulation has been described in the P1 Event-Related Potential (ERP) component, which has been proposed as an index of the early visual response in the visual cortex (Clark et al., 1995; Di Russo et al., 2002; Luck et al., 1990; Natale et al., 2006; Sergent et al., 2005). Devia et al. (2017) reported that the amplitude of the P1 component is larger when the stimulus was fixated after a saccade compared to flashed stimuli (i.e., non-saccade-mediated fixation) in a free-viewing paradigm. They also described earlier latencies of the P1 component, suggesting that the visual cortex activates faster to visual stimuli when they are a consequence of a motor act, such as saccades, compared to visual stimuli that are passively sensed. P1 earlier latency was also observed by Schafer and Marcus (1973) when self-initiated visual stimulus was compared to externally-passively presented stimulus. These studies suggest that self-initiated stimulus can modulate early sensory processing deployed during WM encoding.

A similar effect is observed on attentional neuronal substrates when stimulus is self-initiated. Self-initiated stimulus shows increased amplitude of both P3a and P3b components compared to passive externally paced stimulus (Nittono et al., 2003; Nittono, 2004, 2006). Since P3a is considered to be an index of attentional capture, while P3b reflects the updating memory mechanism (Polich, 2007), this evidence points that self-initiated stimulus enhances attentional processing, through enhancement of both attentional capture and updating mechanisms in memory. These findings suggest that self-initiated stimulus can also modulate WM at a later stage of encoding.

As for effects of self-initiated stimuli in memory tasks, behavioral evidence reported better novel melodies recognition in skilled pianist when the melody was learned in an auditory-motor context (i.e., the pianist performed the melody) than auditory-only (i.e., just listening to melodies) condition (Brown and Palmer, 2012). However, this study did not assess whether this improvement is due to a sensory, attentional or/and memory updating neural modulation. Non-self-initiated WM tasks have revealed that successful stimuli encoding is related to a modulation of attentional indexes such as P3 (Polich, 2007) and P2 (Dunn et al., 1998; Missonnier et al., 2004). Parietal P3 is usually defined as a memory updating mechanism (Polich, 2007), and is related to posterior parietal cortex activity (Knight et al., 1989; Verleger et al., 1994). Classic WM studies have shown that the amplitude of P3 during encoding is an index of successful encoding, such that greater amplitude of P3 during encoding correlates with later successful retrieval (Karis et al., 1984; Fabiani et al., 1986). On the other hand, P2 is conceived as an index of attentional resources used during WM encoding (Dunn et al., 1998; Missonnier et al., 2004). Earlier P2 latency correlates with better WM task performance (Missonnier et al., 2007). Thus, we expect to see a modulation on later attentional processing, reflected in greater amplitudes of P3 and earlier latencies of P2 for self-initiated stimuli compared to externally initiated ones.

Self-initiated tasks usually allow for temporal prediction of the stimuli appearance since the movement that triggers the stimuli is finely coupled in time (i.e., time-locked) to the sensory consequence (Hughes et al., 2012; Schröger et al., 2015). Nevertheless, motor systems also activate in self-initiated tasks, such that both temporal prediction and purely motor activation could be underlying the effects of self-initiated tasks. Temporal prediction, even if it does not occur in the context of self-initiation, refers to a neural mechanism in which the "processing and detection of events are facilitated by minimizing the uncertainty about when they are going to occur" (Arnal and Giraud, 2012). Temporal prediction of externally presented probes enhances WM performance by an attentional modulation (Jin et al., 2020; van Ede et al., 2017; Wilsch et al., 2015), so it possible that self-initiated stimuli during WM tasks could rely on such mechanism. However, if the effect of self-initiated stimuli relies on time-prediction only, then jittering between the motor act and its sensory consequence should abolish those effects. Self-initiated stimuli modulate sensation, even when the stimulus onset cannot be accurately predicted (Bäss et al., 2008; Lange, 2011), suggesting a motor-related mechanism involved in perception in addition to temporal predictive mechanisms. Even more, movements made before and after stimuli presentation (hence, the stimuli are not an immediate consequence of the motor act and are not necessary to the goal accomplishment) can also influence perception (Horváth et al., 2012; Tomassini et al., 2015) and even long-term memory performance (Yebra et al., 2019). This evidence is suggestive of a possible motor-related modulation that is (at least in part) parallel to temporal prediction. Yebra et al. (2019) indicated that the noradrenergic system's engagement mediates this motor-related improvement in long-term memory performance during encoding. Since the noradrenergic system massively modulates brain activity (Berridge and Waterhouse, 2003), it is possible to speculate an influence at both early sensory and attentional encoding processing levels. Moreover, the motor cortex activity, such as the supplementary motor area (SMA), has been proved to modulate mice's visual cortex through anatomically direct connections (Leinweber et al., 2017). Although this has not been confirmed in humans, a hand movement such as a button press that triggers the stimuli onset could modulate the cortical visual processing through SMA-visual cortex connections. If this is true, self-initiated stimuli should yield a modulation in the early visual encoding indexes such as P1 and N1 ERP components during WM encoding. Since SMA is also connected to the cortical regions related to the attentional mechanisms involved in WM, namely posterior parietal cortex (PPC) (Bozkurt et al., 2017; Reep et al., 1994; Wilber et al., 2015) and dorsolateral prefrontal cortex (DLPFC) (Bozkurt et al., 2017; Hasan et al., 2013), it is possible to expect a modulation of attentional processing during WM encoding, indexed by P3and P2 components.

Based on the literature reviewed above, we hypothesized that self-initiated stimuli improve WM encoding. This effect relies on the modulation of visual, attentional, and memory updating processing. To test this hypothesis, 25 healthy adults performed a modified Sternberg WM task (Sternberg, 1966) with three different ways of deploying the stimuli, while EEG activity was recorded. The classic control WM paradigm (Passive (P) condition), consisting of the automatic presentation of the stimuli, was compared to two self-initiated conditions: an Active Coupled condition (AC) and an Active Decoupled condition (AD). Suppose active self-initiated stimuli improve WM through a temporal modulation of visual, attentional, or memory updating processing. In that case, we should find a) better performance in AC than P, b) an effect in encoding the ERPs markers in AC compared to P, and c) a relation between ERP modulation and performance.

Moreover, to test if the self-initiated stimuli effect is based on temporal prediction, an Active Decoupled encoding condition (AD) was designed. AD consisted of the presentation of the stimuli after a random delay (400–600 ms) after the button press, which reduces the temporal predictability of the stimuli onset without affecting the sense of self-control of the stimuli (i.e., agency) (Wen, 2019). In case the modulatory effects of self-initiation rely on time-prediction only, we should find: a) no significant differences in performance and ERP components between AD and P conditions since both conditions lack precise time-prediction of stimuli onset and b) statistically significant difference in performance and ERP components between AC and AD. Our results show an effect of the encoding conditions on both performance and ERP components, with AC yielding better performance and earlier latencies of ERP components compared to both AD and P conditions. Even more, AD condition also presents better performance and earlier latencies of ERP components compared to P condition, suggesting that self-initiation of the stimuli does not rely on the temporal coupling between action and its sensory consequence only.

## 2 Materials and methods

The study methods, experimental protocol and consent was approved by the Ethical Committee in Human's Research of the Medicine Faculty of the Universidad de Chile and followed the Declaration of Helsinki.

### 2.1 Participants

Twenty-six healthy adults (13 females; mean age 23.1 y.o.; range 18–31 y.o.) volunteered and were tested. All the participants were under- or postgraduate students of the Universidad de Chile with non-current or history of neurological, psychiatric, systemic disease, and normal or corrected-to-normal vision. This information was corroborated by anamnesis and the application of the MoCA test (Nasreddine et al., 2005). In the MoCA test, all participants met the inclusion criteria of scoring ≥26 points. As for anamnesis, the exclusion criteria were: a) Cranioencephalic trauma; b) Usage of illegal drugs during the last three months; c) Uncompensated systemic disease (metabolic disease or epilepsy); d) Usage of one or more of these drugs: benzodiazepines, anticonvulsants, metilfenidate, modafinil; and e) Diagnosis of depression or adult attentional deficit disorder. Informed consent was previously read and signed by all the participants. One of the participants was excluded from all analyses due to luminance differences along the recording session. From ERP analyses we disregarded another six subjects because their ERP components were not distinguishable from noise. ERP grand averages were then calculated using only nineteen of the twenty-six participants.

### 2.2 Task

**Figure 1:**
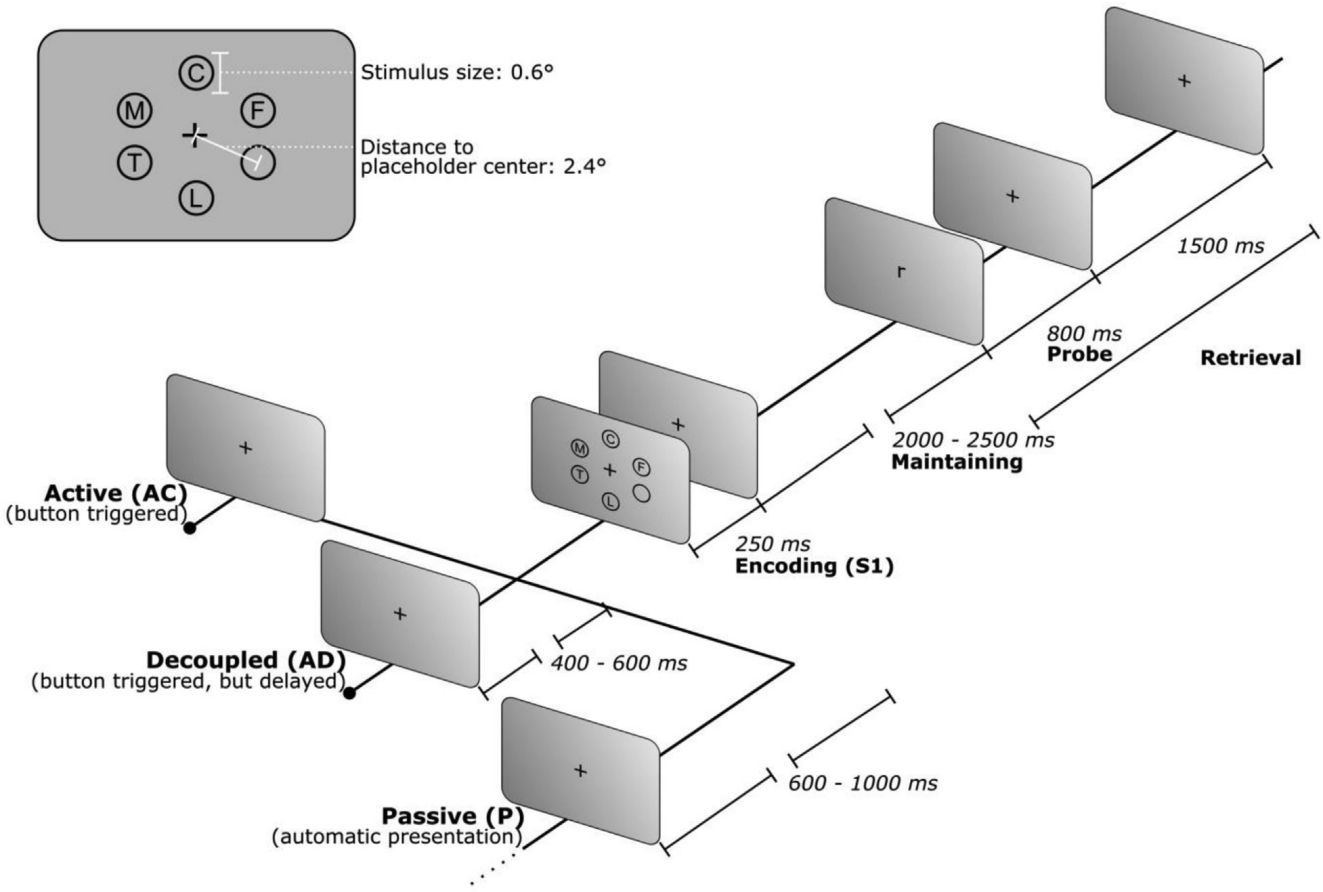
Schematic presentation of the WM task showing the three experimental conditions. Each condition varies only on the pre-encoding stage. Active Coupled condition (AC) starts with a button press, and the stimuli array (S1) is displayed immediately. Active Decoupled condition (AD) also starts with a button press, but the S1 is delayed by a random time between 400 and 600 ms. Finally, Passive condition (P) follows an automatic presentation of S1 at a random time between 600 and 1000 ms following fixation cross onset. The inset shows an example of the S1 array and its visual disposition. (° visual degrees; ms: milliseconds).

Participants engaged in a modified Sternberg working memory task (Sternberg 1966), with three encoding conditions while recording EEG activity. The three conditions differed only in how the stimuli were triggered (whether self-initiated or externally triggered) while sharing the same time course from stimuli presentation until the participants’ answers. Conditions corresponded to an Active Coupled condition (AC), an Active Decoupled condition (AD) and a Passive condition (P). Details of the task scheme are shown in Figure 1.

In all the three conditions, each trial began with an eye-tracking-based drift correction to ensure that participants' eyes positions remain similar in every trial. After drift-correction, a first fixation cross marked the beginning of each trial. The stimuli array (S1) lasted 250 ms (ms). After S1 disappeared, only the central fixation cross remained for a random time between 2000 and 2500 ms (maintaining period). Subsequently, the probe stimulus appeared for 800 ms, followed by the final fixation cross, which lasted 1500 ms (retrieval). Subjects had all the retrieval time to respond (800 ms + 1500 ms = 2300 ms). If the probe was present in the S1 array, participants had to press the joystick's right back button with their right index finger. Conversely, if the probe was absent from set S1, participants had to press the left-back button with their left index finger.

As stated before, to determine if self-initiated stimuli modulate WM, the onset of the S1 set was manipulated, generating three encoding conditions: AC, AD, and P. In the AC condition, we required the participants to fixate their gaze to a cross and to press a frontal button of the joystick (with either of their thumbs). Immediately after the button press, the S1 array appeared on the screen for 250 ms. The AD condition was very similar: participants fixed their gaze to a cross and then pressed a frontal button but, S1 appeared with a random delay (400–600 ms) after the button press. This time-lapse does not disturb the sense of agency of individuals (Wen 2019). Finally, the P condition corresponds to the classic automatic presentation of the stimuli, which appear after a random fixation time of 600–1000 ms. We presented the P condition with random times, due to in real-life WM context, the relevant stimulus is usually randomly presented.

Each condition was presented in separate blocks of 100 trials. Each participant executed all the three blocks, accomplishing a total of 300 trials. The presentation order of the blocks was pseudo-randomized per participant. Instructions were given separately for each condition, at their beginning. Participants were not told about the specific difference between AC and AD conditions to not bias their answers. At the end of each block, participants 12 to 25 determined the task's difficulty. To do so, they rated their global sensation of how secure they made their answers in that particular block (hereafter "reported confidence"). We used a scale of confidence ranging from 1 to 7: 1 corresponded to "not sure of my answers," 4 was neutral confidence, and 7 corresponded to "very sure of my answers." The experimental paradigm was designed in Experiment Builder (SR Research Ltd., Mississauga, Ontario, Canada) and executed in Eyelink 1000 (SR Research Ltd., Mississauga, Ontario, Canada).

### 2.3 Materials

The stimuli were presented in a flat *Viewsonic vx2*753mh*-led* 27 inches screen (23.54 × 13.24 inches; resolution 1920 × 1080 pixels; refresh rate 60 Hz). Subjects seated at a 72 cm distance from the screen. At this distance, 40.35 pixels equals 1 visual degree (°). Images were 45.1° wide and 26.3° high. Subjects kept their chin on a chinrest during the whole experiment to reduce head movements and to maintain a stable distance between the eyes and the screen. We monitored fatigue blinks and eye positions with an Eyelink 1000 (SR Research Ltd., Mississauga, Ontario, Canada) eye-tracking system.

The background was set to gray (127/127/127 RGB) during the whole session to avoid luminance changes. All the stimuli (S1 and probe stimulus) were black consonants. S1 consisted of five capital consonants (except “X” and “Y”) presented simultaneously in a circular array surrounding a fixation cross (Figure 1). The fixation cross appeared at the center of the screen (at pixel coordinates 960,540). Stimuli positions were demarcated by six circles as placeholders (based on Green and Bavelier, 2003; Proksch and Bavelier, 2002). Therefore, one circle was left empty in every trial. To avoid habituation, each of the six circle positions had the same probability of being empty. As for the stimuli's size, consonants were set to a height of 0.6°, while the placeholders' circles had 1° diameter (based on Green and Bavelier, 2003; Proksch and Bavelier, 2002). Placeholders centroids were set at a 2.4° distance from the central fixation cross. Since the parafoveal region has a size of 5.2° (Kolb 1995), this disposition ensured that the stimuli inside the placeholders fit into the parafoveal region. Three hundred S1 sets were created, one for each trial. Each S1 set consisted of five unrepeated consonants chosen randomly by the "rand" function in Matlab©. Consonants "B", "V", and "W" never appeared in the same set S1; the same occurred with "M" and "N". This was done to avoid acoustical or visual associations. As for the probe stimulus, it consisted of a unique lowercase consonant (excluding "x" or "y") presented in the center of the screen (coordinates 962,540). The presence or absence of the probe stimuli on the S1 set had the same probability (50%). Responses were made by pressing a button with either left (probe is not present on S1) or right (probe is present in S1) index finger. A joystick, model Microsoft SideWinder, was used to record the response.

### 2.4 Behavioral data analysis

To assess if self-initiated stimuli do influence WM, we analyzed the encoding condition's effect on accuracy and reaction times (RT). We defined accuracy as the proportion of correct answers (True Positives and True Negatives) for each participant. RT corresponded to the time (in ms) elapsed since the appearance of the probe until the participant's answer. RT was calculated using only correct trials. We tested the effects of the encoding condition on the two dependent variables using two One-way-repeated ANOVA. We computed post hoc tests for each variable with significant effects using a paired-t-test corrected by the Holm-Sidak method. We chose these parametric tests according to results of normality distribution (assessed by Shapiro-Wilk test) and homoscedasticity (assessed by Levene test) of both accuracy and RT (See supplementary Table S1). We also analyzed the reported confidence rating using the Friedman rank sum test. This result can be generalized to the whole sample, since the sub-sample with reported confidence ratings has no statistically significant difference with the sub-sample without reported confidence ratings in terms of accuracy, RT, MoCA results, and age (see supplementary Table S2).

Next, we evaluated the impact of covariates on WM behavior. We identified four variables that could potentially be influencing performance. These covariates are: task learning, block position, uncontrolled pre-stimuli time and total pre-stimuli time. We defined task learning as the change in the probability of making a correct response due to the increasing trial number (from 1 to 100). The block position was a categorical variable, and its value could be the first, second, or third block. We defined the uncontrolled pre-stimuli time as the time before the stimuli onset whose duration was not controlled by the subjects. In the passive condition, the uncontrolled pre-stimuli time was the duration of the first fixation cross. In the AD condition, the uncontrolled pre-stimuli time was the duration of the time delay between the button press and the onset of the S1 set. In the AC condition, the uncontrolled pre-stimuli time was zero ms. The total pre-stimuli time corresponded to the uncontrolled pre-stimuli time plus the time taken by the participants to press the button in the AC and AD conditions; in P condition this value was equal to the uncontrolled pre-stimuli time.

**Figure 2:**
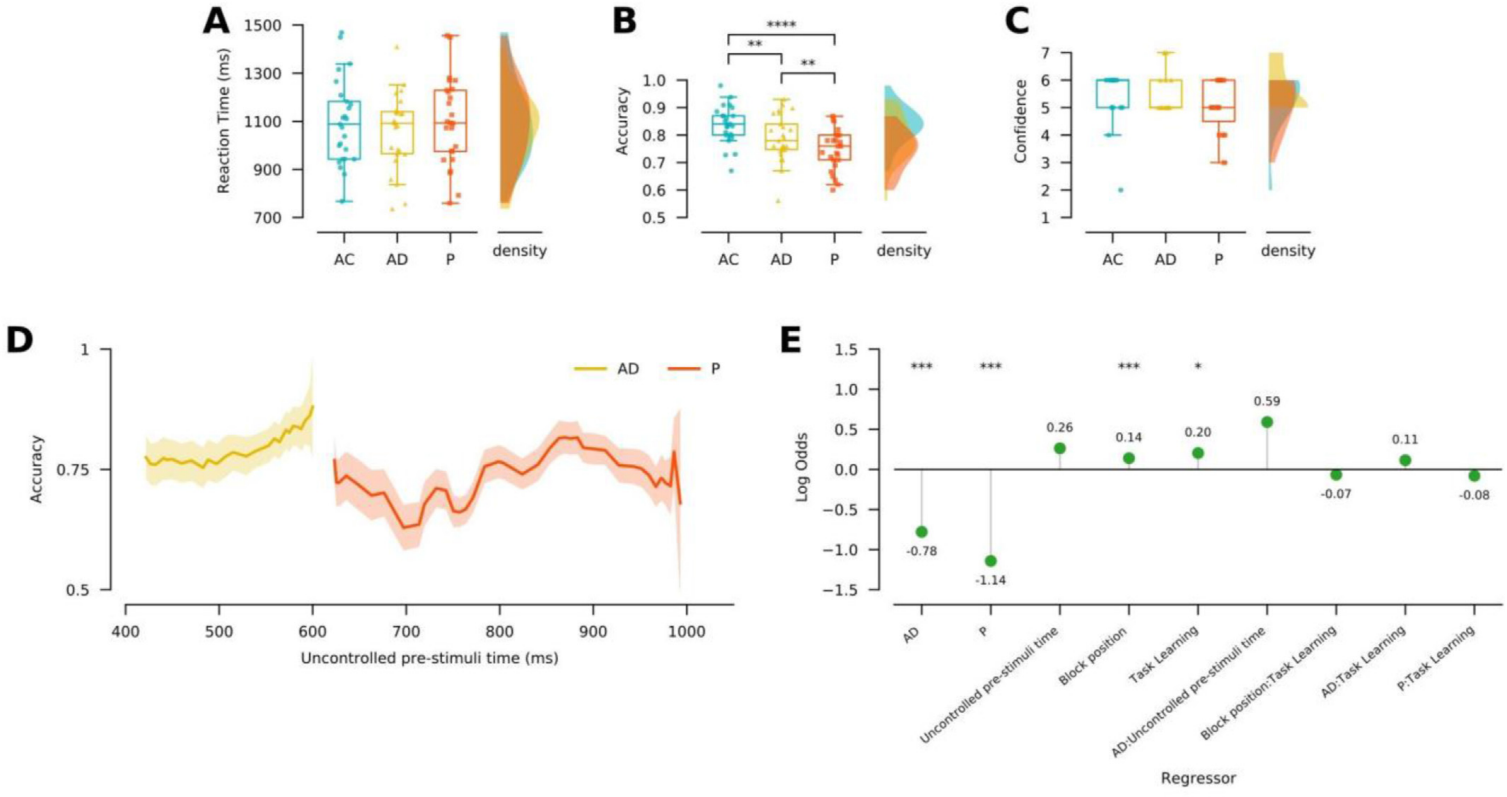
Self-initiated stimuli effect enhances WM performance. (A) Reaction times per encoding condition. Left panel, box plot shows the reaction times in milliseconds (ms) per condition (AC: light blue; AD: yellow; P: orange). Right panel, density plot shows the distributions of the reaction times per encoding condition (n = 25). Dots in box plots represent the value for each participant. Upper limit of the box = 75th percentile; lower limit of the box = 25th percentile; upper whisker = upper limit value; lower whisker = lower limit value; outlier values are shown outside the whiskers. (B) Similar to A, but for accuracy (n = 25). (C) Similar to A, but for reported confidence ratings (n = 15). (D) Accuracy as a function of the uncontrolled pre-stimuli time in the AD and P conditions. The uncontrolled pre-stimuli time of AC is fixed at 0 ms, so it is not shown in the figure. The thick line represents the mean accuracy at the specific time-point. Shaded lines represent the 95% confidence intervals (n = 25). (E) Effects of variables (regressors) over the estimated value of making a correct answer. Colon () indicates interactions between parameters. (∗: p ≤ 0.05; ∗∗: p ≤ 0.01; ∗∗∗: p ≤ 0.001; ∗∗∗∗: p ≤ 0.0001).

Since the stimulus onset time is different for each condition, we first explored the influence of the uncontrolled pre-stimulus time on accuracy. To do so, we first computed the mean accuracy and the 95% confidence interval along the time-series for the AD and P conditions separately (Figure 2D), and performed a Linear Mixed Model (LMM) for each of them. The mean accuracy was calculated in 100 ms moving windows, in steps of 10 ms, from 400 to 600 for AD and 600 to 1000 for P condition. Mean accuracy was first calculated separately for each subject. Grand average accuracy, standard error and CI were then calculated. After that, we performed the LMM analysis on the grand averages.

Next, we wanted to measure the main effect of each of the covariates (as well as their interactions) over accuracy. To do so, we computed six separate binomial General Linear Mixed Model (GLMM) (Moscatelli et al., 2012). We set trial accuracy as the dependent variable and participants as random intercept in all the six models. In two of the six models, we included the encoding conditions as random slope. The six tested models included the following variables as fixed effects: encoding conditions, task learning and block position time (see supplementary Table S3). We standardized all the four independent variables using z-score transformation. To select the best model, we pruned them in a backward way by setting all the possible confounding variables as regressors and then removing the non-significant regressors. We performed parallel models including the uncontrolled pre-stimuli time and the total pre-stimuli time. To select the best model, we applied the Akaike information criterion (AIC).

### 2.5 Electroencephalography and signal pre-processing

We recorded Electroencephalographic (EEG) activity at a 2048 Hz sample rate using a BioSemi Inc. amplifier of 32 active scalp electrodes. We used the Common Mode Sense (CMS) and the Driven Right Leg (DRL) electrodes as ground electrodes. Subjects used head caps to hold the electrodes according to the 10/10 system (Jurcak et al., 2007). Eight external electrodes were set: one for each mastoid and six to record the eye movements (EOG): three around the right orbit and three around the left orbit.

We offline re-referenced the continuous EEG signal to the average activity of the 32 electrodes. Then, we filtered the signal using a FIR symmetric passband filter between 0.5 and 40 Hz with a linear phase. Its design is firwin with a Hamming window (acausal, zero-phase delay, and one-pass forward). The size of the filter was 6.6 s. The transition bandwidth of the filter was 0.5 Hz in the lower frequency limit and 10 Hz in the upper-frequency limit. The passband ripple of the filter was 0.0194 dB and the stopband attenuation was set at 53 dB. We discarded noisy channels by visual inspection and then interpolated using spherical splines. None of the channels of interest (Fz, Pz nor Oz) were interpolated. After that, we performed an Independent Component Analysis (ICA) to determine and eliminate components related to blinks and eye movements. We automatically eliminated segments containing muscle artifacts and other artifacts unrelated to blinks through a 500 μV peak-to-peak rejection threshold. This preprocessing stage was done using MNE-Python (Gramfort 2013).

### 2.6 Event-related potential (ERP) components calculation

We divided the continuous signal into epochs centered on the stimuli's appearance. We set the epoch to 500 ms before and 1000 ms after the stimuli appearance. We rejected noisy epochs using a 250 μV peak-to-peak threshold. We applied a baseline correction of -200 to 0 ms to AD and P epochs, and of −500 to −300 ms to AC epochs. The last, being due to a motor component (most likely the readiness potential) present in the AC condition. We then explored ERP components related to those processes to analyze the influence of self-initiation of the stimuli in early visual, attentional, and memory updating processing. Components of interest were *P100-like, N100-like, P200-like, and P300-like* (hereafter, P1, N1, P2, P3)*.* We used electrode Oz to calculate P1 and N1 components (Mangun and Hillyard, 1991), Fz to calculate the P2 component (Missonnier et al., 2007), and Pz to obtain the P3 component (Johnson, 1993; Polich, 2007). All ERPs' components were computed by averaging correct and cleaned trials. We assessed if ERP components of interest (P1, N1, P2, P3) were distinguishable in each subject averaged data by visual inspection using Fieldtrip Toolbox (Oostenveld et al., 2011). The averaged data of six subjects had no distinguishable ERP components due to noise or alpha oscillations. That data was not used for further EEG analyses, therefore we included nineteen subjects for EEG analyses. Next, we analyzed the amplitudes and latencies of each ERP component. We calculated the amplitude of each component using peak-to-peak values. The peak-to-peak method consisted of calculating the component's absolute peak amplitude in a certain electrode and time-window, then calculating a reference absolute value and subtracting both the values. To determine each component's peak value, we first calculated the latency of that component from the grand-average (average of the ERP across all nineteen subjects). We then calculated the absolute peak amplitude and latency in each subject in a 100 ms window around the grand-average latency for the P1, N1 and P2 component. In the P3 component, the window was set at 200 ms. The reference value for the N1 component corresponded to P1, while the reference values for P2 and P3 were defined as the negative peak appearing immediately before either P2 or P3. We used this approach to calculate amplitude values, since a component near 0 ms appeared in AC condition only, which could influence the peak amplitude values in the AC condition. We calculated the ERP components using MNE-Python (Gramfort 2013).

### 2.7 Event-related potential (ERP) statistical analysis

To test if there was an effect of the encoding condition over amplitude and latency, we performed either one-way ANOVA or Friedman tests (one per ERP component), based on the results of normality and homoscedasticity. The factor corresponded to CONDITIONS in both cases. The rank-sum Wilcoxon or t-test corrected by Holm-Sidak was performed as a Post Hoc test.

To evaluate which neuronal marker is related to the accuracy improvement found in the voluntary self-initiated condition, we used a conditional Classification Tree (CART model). CART models allow us to classify or estimate phenomena with discrete changes (Hothorn et al., 2006a; 2006b; Strobl et al., 2009). For this particular CART model, we selected the Monte Carlo Method, with 1000 resampling to estimate tree splits' significance using an alpha of 0.05. Additionally, to avoid overfitting the model, the amount of observation per leaf was limited to 20% of the total observations. The partitioning variables used to characterize the accuracy corresponded to the amplitudes and latencies of P1, N1, P2, and P3 components. The CART model yielded P2 latency as the electrophysiological variable that better explains the accuracy improvement (see Results). Next, we seeked to test if the relationship between the latency of P2 and accuracy was dependent on the encoding condition. To do so, we performed a Linear Mixed Model (LMM). The model included accuracy as a dependent variable, the interaction between P2 latency and the encoding conditions as a fixed effect, and subjects as random effect variable.

Then, we used a second CART model to assess which electrophysiological variable distinguishes better among encoding conditions. The partitioning variables used to characterize the encoding conditions were accuracy, RT, and the amplitudes and latencies of P1, N1, P2, and P3 components.

We performed all statistical computations using RStudio (Rstudio Team, 2016). Rstudio libraries used were EZ (v4.4-0) (Lawrence, 2016) to run ANOVA tests, car (v3.0-2) (Fox and Weisberg, 2014) to run Levene test, party (v1.3-5) (Hothorn et al., 2015) to run CART models, and lme4 (v1.1-21) (Bates et al., 2015) to compute GLMM and LMM.

## 3 Results

We hypothesized that self-initiated stimuli presentation improves WM performance through the temporal modulation of visual, attentional, and memory updating processing. To evaluate how self-initiated stimuli would affect WM encoding, twenty-six participants performed a Sternberg working memory task with three different stimuli's encoding conditions. In the Active Coupled (AC) condition, the stimuli were presented immediately after the participants pressed a button. In the Active Decoupled condition (AD), stimuli were presented with a delay of 400–600 ms after the button press, which allowed us to test whether possible effects of self-initiated stimuli are tied to temporal precision of stimuli onset. Finally, these two self-initiated conditions were contrasted with the passive presentation of the stimuli (P), where stimuli were automatically presented between 600-1000 ms after the first fixation onset. Each encoding condition consisted of one block of 100 trials so that each subject executed three separate blocks (one per encoding condition) (See Materials and Methods).

We tested the proposed hypothesis by two approaches: i) Is behavior modulated by these three experimental conditions? and ii) Does self-initiation modulate early visual, attentional, and memory updating processing during encoding?

### 3.1 Voluntary self-initiation of the stimuli enhances WM performance

To explore whether self-initiated stimuli presentation modulates WM mechanisms, we tested the effect of encoding conditions (AC, AD, and P) on performance (RT and accuracy). We assessed this in twenty-five of the twenty-six recruited subjects (see Materials and Methods, participants). Table 1 shows median and confidence intervals for RT and accuracy, specified per encoding condition. The ANOVA results reveal a main effect of encoding conditions on accuracy (F_(2,48)_ = 25.67, p = 2.6e-08; η^2^_G_ = 0.2). No encoding condition effects were found on RT (F_(2,48)_ = 1.45, p = 0.244) (Figure 2). These results reveal that self-initiated stimuli modulate accuracy without significantly affecting RT (Figure 2A).

**Table 1:** Descriptive and analytical statistics results.

			Descriptive statistics Median [Confidence Interval]			Analytical statistics p-value (size effect)		
			AC	AD	P	AC-P	AC-AD	AD-P
**Behavioral results (n = 25)**	**RT (ms)**		1088.36 [1026.77–1165.67]	1092.22 [995.23–1119.13]	1093.21 [1028.33–1171.42]	n.s.	n.s.	n.s.
	**Acc**		0.84 [0.81–0.86]	0.78 [0.76–0.82]	0.76 [0.72–0.77]	5.9e − 08 (1.64)	0.003 (0.65)	0.003 (0.71)
	**Conf**		6 [4.84–5.7]	5 [5.28–5.92]	5 [4.68–5.44]	n.s.	n.s.	n.s.
**ERP results (n = 19)**	**Amp. (μV)**	**P1–N1**	8.58 [7.65–10.29]	9.79 [9.19–10.99]	8.56 [8.38–10.19]	n.s.	n.s.	n.s.
		**P2**	5.92 [5.17–7.81]	5.81 [5.37–7.16]	5.95 [5.32–7.13]	n.s.	n.s.	n.s.
		**P3**	6.52 [5.23–6.67]	6.03 [5.12–7.03]	6.39 [5.08–8.46]	n.s.	n.s.	n.s.
	**Lat. (ms)**	**P1**	118.16 [109.41–120.39]	129.88 [118.88–129.74]	134.27 [125.33–137.26]	1.1e − 05 (−1)	0.014 (−0.73)	0.018 (−0.63)
		**N1**	171.39 [168.76–176.69]	187.5 [183.72–188.87]	187.01 [184.86–192.19]	1.2e − 08 (−2.41)	8.01e − 07 (−1.69)	n.s.
		**P2**	169.43 [165.76–175.83]	183.59 [182.05–189.05]	191.41 [186.45–193.85]	9.3e − 13 (−1.77)	7.9e − 09 (−1.2)	0.001 (−0.66)
		**P3**	360.84 [345.28–393.77]	366.21 [357.51–411.11]	416.99 [373.02–427.05]	0.0024 (−0.91)	0.009 (−0.72)	n.s.

RT = reaction times; Acc. = accuracy; Conf = reported confidence ratings; Amp. = amplitude; Lat. = latency; size effect (Cohen's d or r); n.s. = non significant.

To assess if self-initiated stimuli presentation enhances WM performance, we executed a post hoc test, analyzing the accuracy difference between AC (median = 0.84; 95% confidence interval (C.I.) = [0.81–0.86]) and P conditions (median = 0.84; 95% C.I. = [0.81–0.86]). Paired t-tests corrected by the Holm-Sidak method show that AC has better accuracy than P condition (t_(24)_ = 8.21; p = 5.9e − 08, Cohen's d = 1.64). To further investigate if the timing between motor acts and its sensory consequences is relevant to the self-initiation effect, we compared the accuracy between AC and AD conditions (AD median = 0.78; 95% C.I. = [0.76–0.82]). Paired t-tests corrected by Holm-Sidak method also confirm better accuracy in AC compared to AD condition (t_(24)_ = 3.25; p = 0.003, Cohen's d = 0.65). Noteworthy, when subjects engage in a passive WM task, their accuracy is poorer when compared to both active conditions (AD compared to P: t_(24)_ = 3.56, p = 0.003, Cohen's d = 0.71). This indicates the impact of self-initiated stimuli on WM performance, which seems to be partially linked to action and task-relevant stimuli coupling. A remarkable point is that these accuracy effects are not explained by differences in the perceived difficulty of the task since there are no statistically significant differences in their reported confidence, rated by the participant at the end of each condition block (χ ^2^_(2, N=14)_ = 1.47; p = 0.478) (Figure 2C).

We then explored the impact of other cognitive factors on performance. Since the stimuli onset time is variable between conditions, we first assessed the influence of this time on accuracy. Figure 2D shows the mean accuracy and 95% C.I. in function of the uncontrolled pre-stimuli time (see Materials and Methods, behavioral data analysis). The AC condition was excluded from this analysis, since in this condition stimuli onset always appeared simultaneously with the button press. Then, we performed a Linear Mixed Models (LMM) separately for AD and P. Both models included accuracy as the dependent variable, uncontrolled pre-stimuli time as fixed effect variable, and subjects as random effect variable. Results yielded a significant effect of uncontrolled pre stimuli time on accuracy in the AD condition only (AD:*β* = 1.42e − 0.1 ± 5.35e − 02, t = 2.67, p = 0.008; P: *β* = 4.422e − 0.2 ± 2.96e − 02, t = 1.49, p = 0.136). These results show that the uncontrolled pre-stimuli time influences AD accuracy and, therefore, could be a good predictor of accuracy.

To prove this statement and the effect of other potential predictors of the accuracy, we performed a General Linear Mixed Model (GLMM), including encoding condition, uncontrolled pre-stimuli time as well as two other covariates (see Materials and Methods, behavioral data analysis). These covariates included task learning (i.e., the index of trial within the block) and block position. This model yielded the best AIC compared to other models tested (supplementary Table S2). The model results show a significant effect in three of the independent variables assessed: task learning (***β*** = 0.20 ± 0.09, z = 2.25, p = 0.024), block position (***β*** = 0.139 ± 0.04, z = 3.91, p = 9.1e − 05), and the encoding conditions (AD condition: ***β*** = −0.779 ± 0.195, z = −3.99, p = 6.55e − 05; P condition: ***β*** = −1.142 ± 0.33, z = −3.45, p = 5.4e − 04) (Figure 2D). On the other hand, results yield a non-significant trend for the uncontrolled pre-stimuli time (***β*** = 0.263 ± 0.136, z = 1.92, p = 0.054). This implies that, when other predictors are also considered, the probability of making a correct response does not significantly depend on the time that the stimuli take to appear in the AD and P conditions. We find a non-significant trend for the interaction between uncontrolled pre-stimuli time and AD condition (***β*** = 0.59 ± 0.314, z = 1.88 p = 0.06). We did not find a significant interaction between task learning and block position (***β*** = −0.065 ± 0.035, z = −1.84, p = 0.065), nor interaction between task learning and tasks (Task Learning and AD condition: ***β*** = 0.114 ± 0.07, z = 1.55, p = 0.12; Task Learning and P condition: ***β*** = −0.080 ± 0.719, z = −1.11, p = 0.26). These GLMM results corroborate the previous ANOVA analysis, indicating a significant decrease in the probability of making a correct response when subjects are engaged in the AD or the P conditions compared to AC condition. The probability of making a correct response falls 0.779 log-odds points in the AD condition, and the P condition's 1.142 log-odds point. Alongside the task learning effect, results show that the probability of making a correct response rises in 0.2 log-odds points with each trial (from 1 to 100) within one block. Results also yield no interaction between task learning and encoding conditions, showing that task learning has statistically no different effect on all three conditions. Likewise, the model reveals that the probability of answering correctly increases in 0.139 log odds with the block position. Like in task learning, the effect of the block position is also independent of the encoding condition. Finally, our results show that the differences in the onset stimuli time that is not under the control of the subject impacts non-significantly on accuracy, with a trend to increase the probability of making a correct answer in 0.263 log-odds. Altogether, GLMM yields other factors such as task learning and block position that influence performance alongside the encoding conditions effects (Figure 2E).

In summary, behavioral analyses show that coupled self-initiation of the stimuli in a WM task increases the probability of performing correctly, even though other timings and learning factors also modulate this probability. Remarkably, self-initiation improves performance, even when the time between cue onset is less favorable. In other words, even when longer uncontrolled pre-stimuli times correlates with higher accuracy scores, active conditions (whose uncontrolled pre-stimuli times are shorter than passive) perform better than passive.

### 3.2 Voluntary self-initiation of the stimuli accelerates N1 and P2 during WM encoding

**Figure 3:**
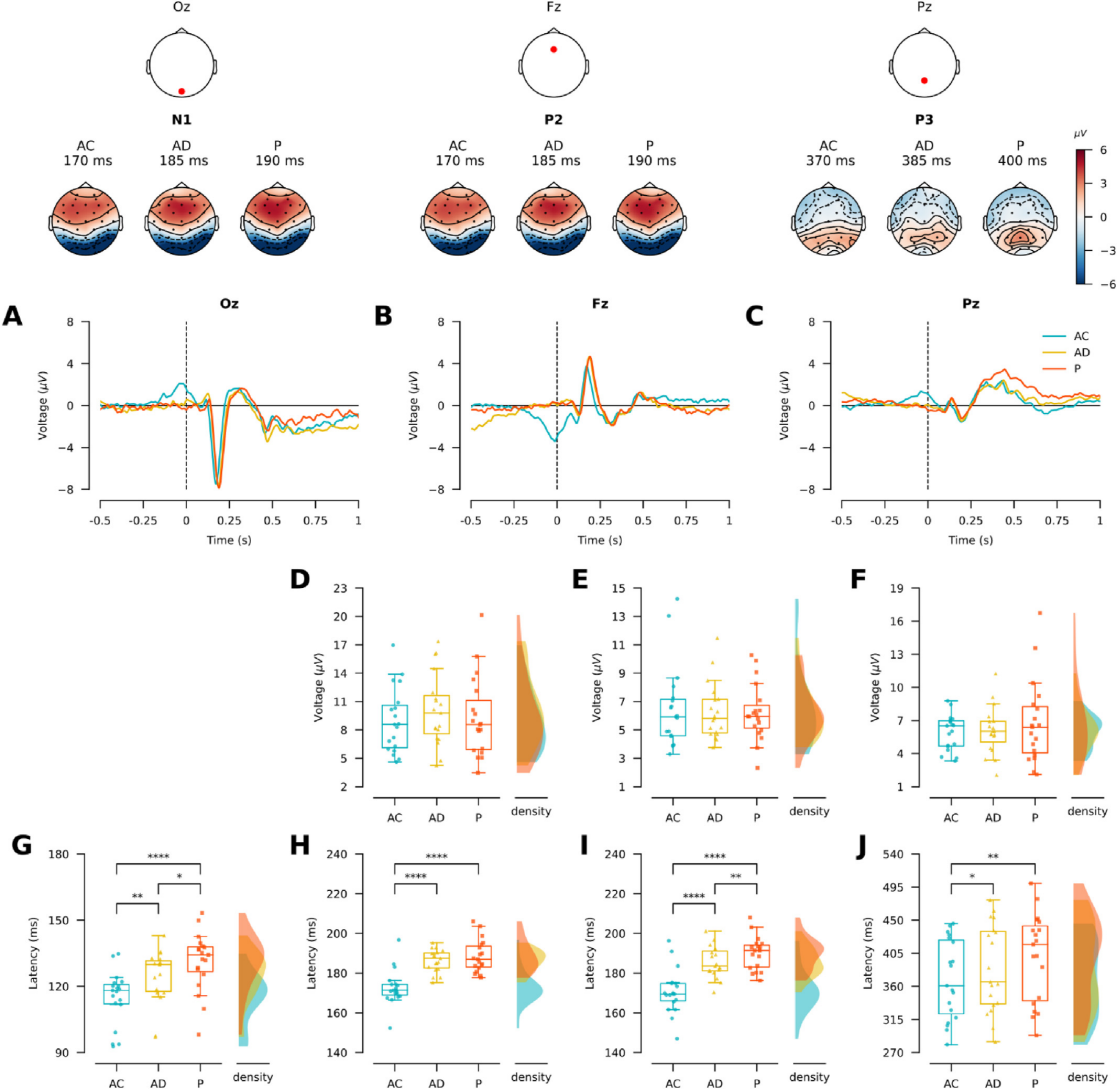
Self-initiated stimuli effect modulates early visual and attentional ERPs indexes. (A) Upper panel Topographical plots of the indicated times. Dots represent modeled electrode positions; red: positive voltage; blue: negative voltage; values in μV. Bottom panel. ERP grand average (n = 19 subjects) evoked by stimuli presentation (t = 0 ms, dotted line) in electrode Oz, per conditions. Only correct trials are included. (B) Similar to A, but for electrode Fz (n = 19). (C) Similar to A, but for electrode Pz (n = 19). (D) Left panel, box plot shows the peak-to-peak voltage (μV) of the P1–N1 component, per condition. Right panel, density plot shows the distributions of peak-to-peak voltage of P1–N1 per encoding condition. (E) Similar to D, but for P2 peak-to-peak amplitude. (F) Similar to D, but for P3 peak-to-peak amplitude. (G) Similar to D, but for P1 latency. (H) Similar to D, but for N1 latency. (I) Similar to D, but for P2 latency. (J) Similar to D, but for P3 latency. (∗: p ≤ 0.05; ∗∗: p ≤ 0.01; ∗∗∗∗: p ≤ 0.0001).

We then assessed whether self-initiated stimuli presentation impacts visual, attentional, and memory updating processing during WM encoding. To do so, the amplitudes and latencies of related ERP indexes were analyzed (see Material and Methods: Participants). P1 component is a known marker of lateral extraestriate visual cortex (Clark et al., 1995); N1 component, proposed as an index of visual discrimination related to the visual cortex (Vogel and Luck, 2000); P2 component reflects attentional processing of stimuli in WM (Dunn et al., 1998); and P3 component is a marker of mental revision of the stimuli (Polich, 2007). The ERP's grand averages for correct trials of 19 subjects are shown in Figure 3, specified per encoding conditions (6 subjects were disregarded for further analyzes, details in Materials and Methods).

The ANOVA results reveal a significant effect of the encoding conditions on the latency of the N1 (F_(2,36)_ = 52.43, p = 2.16e − 11; η^2^_G_ = 0.468) and the P2 components (F_(2,36)_ = 36.39, p = 2.262e − 09, η^2^_G_ = 0.459). Friedman rank sum test yield a significant effect of the encoding conditions on P1 latency (χ^2^_(2, N = 19)_ = 23.38, p = 8.34e-06, ω_kendall_ = 0.6869) and P3 latency (χ^2^_(2, N = 19)_ = 10.91, p = 0.004, ω_kendall_ = 0.893). No statistically significant effect of encoding conditions was found on peak-to-peak amplitude of all the components analyzed (P1–N1: χ^2^_(2, N = 19)_ = 5.05, p = 0.079; P2: χ^2^_(2, N = 19)_ = 0.32, p = 0.853; P3: χ^2^_(2, N = 19)_ = 0.74, p = 0.691). These results suggest a modulatory effect of the encoding conditions on the temporal domain of sensory, attentional processes and memory updating mechanisms. In Figure 3D, E, F, the box plots show the peak-to-peak voltages (μV) and its distributions for the P1–N1, P2, and P3 components, per condition, while in Figure 3G, H, I, J we show values and distributions of the component latency for each condition, for the P1, N1, P2, and P3 components, respectively.

If self-initiated stimuli accelerate sensory, attentional and memory updating processing of WM, self-initiated conditions (AC and AD) should show earlier latencies compared to passive condition. Our results yield that the subjects have earlier latencies in AC compared to P condition in P1 (rank sum Wilcoxon test corrected by Holm-Sidak: V = 0; p = 1.1e − 05; r = −1), N1 (paired t-test corrected by holm: t_(18)_ = −10.51; p = 1.2e − 08, Cohen's d = −2.41), P2 (t_(18)_ = −7.71; p = 9.3e − 13; Cohen's d = −1.77) and P3 (V = 8: p = 0.0024, r = −0.91). On the other hand, P condition has the latest latency in P1 (AD/P: V = 35.5, p = 0.018, r = −0.63) and P2 (AD/P: t_(18)_ = −2.87; p = 0.001, Cohen's d = −0.66) components. Nevertheless, paired tests corrected by Holm-Sidak show no statistical differences between AD and P conditions in the N1 component (t_(18)_ = −1.33; p = 0.2), and in P3 component (V = 60.5; p 0.17). These results suggest an effect of voluntary self-initiation of the stimuli on sensory, attentional memory updating processing, which is stronger when the voluntary action is temporally coupled to the task-relevant sensory consequence.

To further examine if the temporal coupling between motor acts and its sensory consequences is important to the self-initiation effect, we analyzed the latency differences between AC and AD conditions. Paired tests corrected by Holm-Sidak shows earlier latencies of AC compared to AD in P1 (rank sum Wilcoxon: V = 23; p = 0.014, r = −0.73), N1 (t-test: t_(18)_ = −7.35; p = 8.01e − 07, Cohen's d = −1.69), P2 (t-test: t_(18)_ = −5.22; p = 7.9e − 09, Cohen's d = −1.2) and P3 (rank sum Wilcoxon: V = 27; p = 0.009, r = −0.72). As in behavioral analysis, these ERP results suggest that the self-initiated stimuli effect is partially linked to the temporal coupling between action and the task-relevant sensory consequence.

Note that in the AC condition, all the three electrodes plotted: Oz, Fz, and Pz (Figure 3A, B, C) show a component around stimuli presentation. We interpret this component as a neural marker of the motor system activity related to the button press, as it has similar latency and spatial distribution to the Readiness Potential (e.g. Travers et al., 2020). Moreover, it is improbable that this component corresponds to a Visually Evoked Potential (VEP), since the first component of the VEP corresponds to the N1 component, which peaks around 70 ms after the stimulus onset (Creel, 2019). Consistently, this component is also present in AD condition, but only when ERPs are locked to the button press instead of stimuli presentation (see supplementary Figure S1). Therefore, peak-to-peak amplitudes were used to control for overlapping slow components, considering that motor potentials overlapped to some degree with the components of interest in the AC and AD conditions.

In summary, these results support that voluntary self-initiation modulates the temporal domain of the neural mechanisms underlying the visual (P1 and N1 components), the attentional processes (P2 component) and the memory updating mechanism (P3 component) engaged during encoding in WM. Moreover, no significant effects on peak-to-peak amplitudes were observed. Results also show that delays between movements and their sensory consequences (as reflected in AD compared to AC condition) do not yield the same effect on encoding processing. Nevertheless, on comparing AD to passive condition, the latencies of P1 and P2 components appear earlier, suggesting that voluntary motor acts modulate WM, even if they are not coupled to the task-relevant stimuli, nor can they finely predict the stimuli onset time.

### 3.3 P2 acceleration better explains WM accuracy improvement

We then aimed to investigate if earlier visual and/or attentional processing are related to the accuracy improvement found in the voluntary self-initiated condition. To assess this, we performed a conditional Classification Tree (CART model) (Strobl et al., 2009). The partitioning variables used to characterize the accuracy corresponded to the amplitudes and latencies of P1, N1, P2, and P3 components.

**Figure 4:**
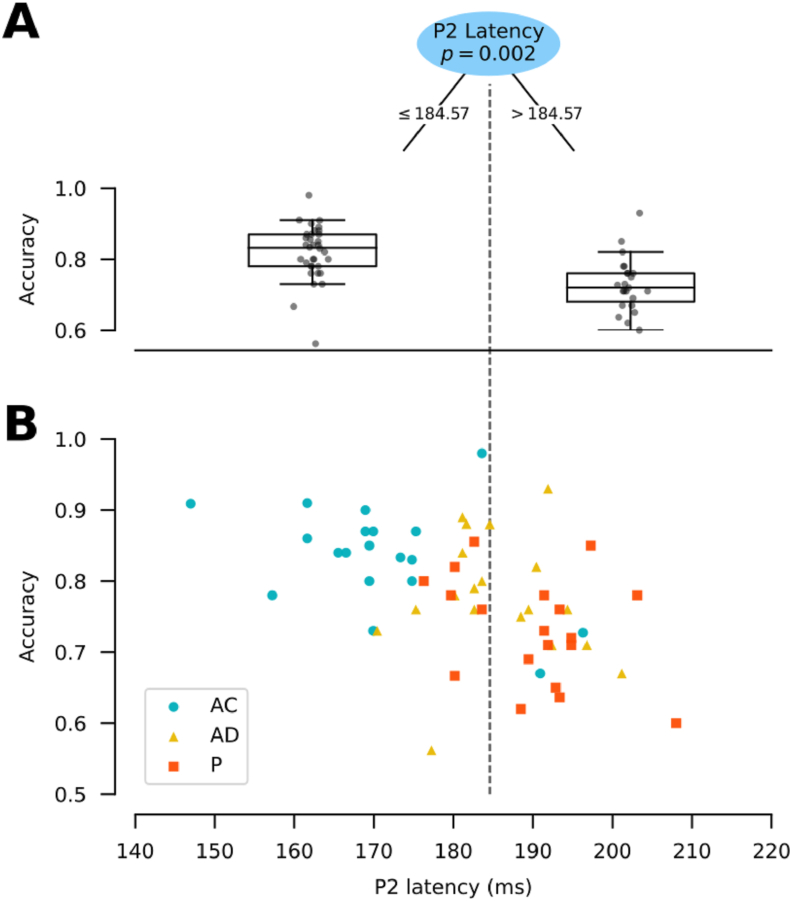
P2 distinguishes between higher and lower accuracy. (A) Accuracy distributions box plots (y-axis) attributed by the CART node based on P2 latency (recorded at Fz electrode), with a split at 184.57 ms (top). The left box plot represents the accuracy distribution associated with P2 latencies earlier than the split. Conversely, the right box plot represents the accuracy distribution associated with P2 latencies later than the split. The number of cases per condition is equal to 19. (B) Scatterplot of accuracy (y-axis) as a function of the latency of the P2 component (x-axis), depicted by the condition (blue circles = AC; yellow triangles = AD; orange squares = P). The discontinuous vertical dashed line represents the split value of the CART model (184.57 ms). Each mark (whether circle, square or star) represents one participant (n = 19 per encoding condition).

According to the CART model result, accuracy can be divided into two groups, higher performance (median = 0.82) and lower performance (median = 0.733), according to the latency of the P2 component (Figure 4A). When the P2 component appears at 184.57 ms or earlier, accuracy is higher. On the contrary, if the P2 component appears later than 184.57 ms, accuracy is lower. No other ERP component yielded significance. This result suggests that accelerated attentional processing is a mechanism involved in WM behavioral improvement.

Given that the above-mentioned result occurs independently from the encoding conditions, we then evaluated if this outcome persists when it is examined by the encoding conditions. P2 latencies from the AC condition (median = 170.79 ± 11.19 ms) show higher accuracy values (Figure 4B). On the contrary, P2 latencies greater than 184.57 ms (found mostly in the AD and P conditions) display lower accuracy (Figure 4B). To ascertain if the observed relationship between P2 and accuracy depends on the encoding conditions, we designed a Linear Mixed Model (LMM). This model included Accuracy as the dependent variable, the interaction between P2 latency and the encoding conditions as the fixed effect variable and subjects as random effect variable. The model indicates that there is not a significant effect of P2 latency on accuracy (β = 2.045e − 04 ± 1.09e − 03, t = 1.88, p = 0.852).

In summary, our results show that P2 latency is inversely related to WM accuracy, with shorter latencies corresponding to better outcomes. Moreover, this relation is dependent on the encoding condition, which is consistent with our ANOVA results showing a task effect on accuracy. This suggests that acceleration of attentional processing is a mechanism related to the WM improvement in voluntary self-initiated conditions.

### 3.4 Earlier visual processing is a marker of a coupled active phenomenon

Next, we explored whether the earlier deploying time of visual and/or attentional processing provides reliable markers of voluntary self-initiation of the stimuli. To do so, we performed a CART model to test how reliably we can estimate the encoding condition of a trial from the latency of its associated ERP components. Since CART models are ideal for classifying or evaluating discrete state variables (Strobl et al., 2009), by using this method, it is assumed that initiating the trial in a coupled way would deploy a phenomenon presumably absent during decoupled or passive conditions. The partitioning variables used to characterize the encoding conditions were accuracy, RT, and the amplitudes and latencies of P1, N1, P2, and P3 components.

**Figure 5:**
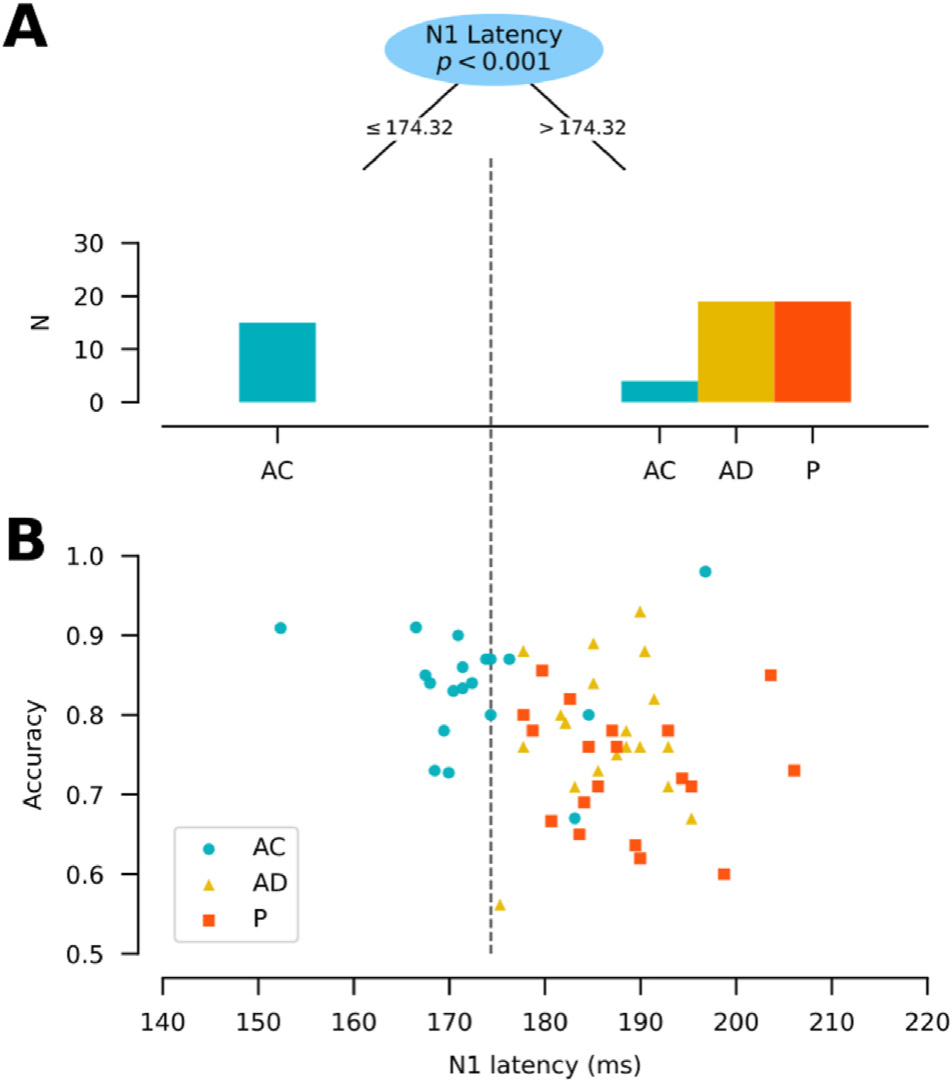
N1 better distinguishes encoding conditions. (A) Histograms of the number of participants per encoding condition (y-axis) attributed by the CART node based on N1 latency (recorded at Oz electrode), with a split at 174.32 ms (top). The left histogram represents the number of cases at latencies equal/earlier than the split. Conversely, the right histogram represents the number of cases at latencies later than the split. The number of cases per condition is equal to 19. (B) Scatterplot of accuracy (y-axis) as a function of the latency of the N1 component (x-axis), depicted by the condition (blue circles = AC; yellow triangles = AD; orange squares = P). The discontinuous vertical dashed line represents the split value of the CART model (174.32 ms). Each mark (whether circle, triangle or square) represents one participant (n = 19 per encoding condition).

CART model result shows that the latency of the N1 component better distinguishes between encoding conditions, using a cutoff of 174.316 ms (p < 0.001) (See Figure 5A). No other ERP variable had a significant effect according to the model. Latencies below 174 ms corresponded exclusively to the AC condition (AC median = 172.75 ± 8.81 ms), while later N1 latencies tended to be found in AD (median = 186.34 ± 5.62 ms) and P conditions (median = 188.53 ± 8.17 ms). This result suggests that earlier visual discrimination processing is related to a coupled self-initiated encoding, and presumably absent in a decoupled or passive one.

Since earlier N1 latency is the variable that better distinguishes AC, and that AC is also the condition that shows better accuracy (See Figure 2B), we analyzed if N1 latency can explain the performance improvement by itself or if it is dependent on the encoding conditions. When contrasted with accuracy, AC's N1 latencies (≤174 ms) tend to show higher accuracy values (Figure 5B). On the other hand, N1 latencies later than 174 ms (mostly compound of AD and P conditions values) tend to show worse accuracy with later latency values (Figure 5B). Our findings show that N1 latency is the variable that better distinguishes a coupled self-initiated encoding process from a decoupled or a passive one, and that N1 latency does not significantly mediate WM improvement.

## 4 Discussion

The current study assesses whether self-initiated stimuli presentation modulates WM encoding through temporal modulation of visual and attentional processing. This was investigated by evaluating the influence of active self-initiated stimuli on behavior in a Sternberg working memory task while concurrently measuring ERPs’ encoding components widely used as indexes of visual (P1 and N1; Clark et al., 1995; Vogel and Luck, 2000), attentional (P2; Dunn et al., 1998) activity, and memory updating mechanisms (P3; Polich, 2007).

### 4.1 Self-initiation enhances WM performance and accelerates visual, attentional and memory updating processing

Our results show that voluntarily initiating the stimuli's onset leads to a performance enhancement in a WM task and to an acceleration in the neural indexes of visual, attentional and memory updating processes. Our findings are consistent with previous research pointing to an improvement of auditory recognition memory of self-initiated compared to externally initiated melodies (Brown and Palmer, 2012). Our data also support that behavioral enhancement is not explained as differences in task difficulty since there is no distinction in the reported confidence among the three encoding conditions. Alongside this, there is no statistically significant difference in reaction times (RT), which is classically modulated by task difficulty (Nickerson, 1972; Pins and Bonnet, 1997; Schneider and Anderson, 2011). Our findings instead suggest a role of temporal predictability and/or motor systems modulation on cognition.

It has been widely proposed that motor actions can predict the timing of the stimulus onset (Arnal and Giraud, 2012; Fujioka et al., 2012; Saleh et al., 2010; Stenner et al., 2015). Motor regions such as the primary sensorimotor cortex (SM1), supplementary motor area (SMA), and cerebellum have been proved to be sensitive to auditory regularities even when auditory stimuli are not under the focus of attention, suggesting the participation of these cortices on the temporal prediction of stimuli (Fujioka et al., 2012). While temporal prediction of the stimuli mediated by both overt and covert movements tend to reduce sensation and its neural correlates (Blakemore et al., 1999; Klaffehn et al., 2019; Lange, 2011; Mifsud et al., 2018; Voss et al., 2008; Wolpe et al., 2018; but for a contradictory effect, see Rohenkohl et al., 2014), no evidence of such reduction in sensory components' amplitude was found in the current work (See Figure 3). On the contrary, our results show an acceleration on visual processing marked by P1 and N1. These seemingly contradictory results could be explained because the conventional self-initiated stimulus paradigms usually prove not just temporal predictability, but also the stimulus-identity predictability. Therefore, the subjects can reliably predict “when” and “what” the movement's effect, which in turn produces the sensory attenuation effect. In our task, however, subjects could not predict the identity of the stimuli since it changed on every trial. This could explain why our results show no sensory attenuation, but a facilitation of the perceptual processing.

Our results are more in line with previous studies suggesting that motor-mediated temporal prediction facilitates sensory processing (Devia et al., 2017; Ito et al., 2011; Schafer and Marcus (1973); Schwarz et al., 2018). Moreover, when temporal prediction interacts with both bottom-up and top-down attentional orientation mechanisms, time prediction boosts performance and its neural correlates (Kaiser and Schütz-Bosbach 2018; Kok et al., 2012; Marchant and Driver 2013; Miniussi et al., 1999; Morillon et al., 2016). Likewise, temporal prediction of externally presented probes enhances WM performance by an attentional modulation (Jin et al., 2020; van Ede et al., 2017; Wilsch et al., 2015), highlighting the relevance of temporal prediction in WM contexts. Consistent with these reports, our findings show that time-locked motor-mediated triggering of the stimuli (AC condition) is associated with both attentional acceleration and performance improvement in a WM task, which could be explained by the temporal prediction produced by the motor action.

It is also known that the time between a cue and a stimulus onset is another factor that modulates stimulus processing through temporal expectations. Longer pre-stimulus times lead to better discrimination due to the hazard function, where stimuli probability of presentation increases with time due it has not currently appeared (Duncan et al., 1994; Müller et al., 1998; Van Ede et al., 2012). In agreement, we found that longer pre-stimulus times (the cue being the fixation offset or the button press) lead to performance improvement within AD and P encoding conditions. Here, time plays an opposing role to that of the encoding condition itself: pre-stimulus time was longer in the worst-performing condition (P) condition and shorter in the best-performing condition (AC, where it was null). This suggests that the impact of self-initiating the stimuli could be even greater than what is seen in the accuracy results, as it was mitigated by the longer pre-stimulus time for the condition with no self-initiation. Nevertheless, our current design cannot finely determine this point since AD and P conditions lack equal delay times.

If self-initiated stimuli effect is solely based on temporal predictability, it should be suppressed under active-unpredictable situations. Our results suggest that the self-initiated stimuli effect is maintained when the stimuli randomly appear after the button press, even though it is attenuated with respect to the fully predictable condition. These results indicate that temporal predictability seems to not be the only mechanism involved in the self-initiation effect. Embodied cognition perspective states that subjects' bodies, particularly their motor systems, influence cognition (O'Regan and Noë 2001; Varela et al., 2016). In agreement, it has been shown that the motor system modulates perception, even though the motor behavior is not relevant to the current task (Tomassini et al., 2015). Concordantly, auditory sensory response is significantly attenuated for self-initiated tones compared to equally predictable externally generated tones (Klaffehn et al., 2019). Moreover, self-initiated stimuli attenuate sensation, even when the timing cannot be accurately predicted, suggesting a motor-related and prediction-independent mechanism involved in perception (Bäss et al., 2008; Lange, 2011), suggesting that sensory attenuation effect is not solely due to temporal predictability. Evidence also shows that unrelated button pressing improves long-term memory encoding, whether the action is time-locked to stimulus onset or not (Yebra et al., 2019). Therefore, our results also suggest a parallel non-predictive modulation of motor systems over WM, which needs to be settled by equating the delay times in self- and externally initiated stimuli. This modulation is possibly related to the recruitment of catecholaminergic pathways needed to be investigated in future studies.

### 4.2 WM improvement in self-initiation of the stimuli onset correlates with attentional acceleration

Our results show that self-initiated stimuli effect accelerates visual perception (indexed by P1, N1; Clark et al., 1995; Vogel and Luck, 2000), attention (indexed by P2; Dunn et al., 1998) and mental revision of WM stimuli (indexed by P3; Polich, 2007). Although classic WM studies have shown that the amplitude of P3 during encoding is an index of successful encoding (Karis et al., 1984; Fabiani et al., 1986), no such correlation was found in our study. Conversely, our results relate WM performance with attentional modulation, which corroborates previous investigations (Missonnier et al., 2007; van Ede et al., 2017; Wilsch et al., 2015). This is also in line with theoretical proposals stating that attentional processing is a critical WM component (Cowan, 2005).

It is noteworthy that no effects on P1, N1, P2, or P3 amplitudes were found, suggesting that self-initiation effects are not based on modulating the number of neural populations required by the task but on influencing the time domain in which encoding is deployed. It could be argued that the motor potential seen around stimuli onset in the AC condition could have biased amplitude values, since AC had different voltage value at zero epoch time. However, to minimize the possible effect of this component we used peak-to-peak values rather than absolute values. In this sense, we used reference value (in this case, another peak) to avoid artificial differences between the amplitudes.

Altogether, our data suggests that self-initiated stimuli effect enhances WM performance and engages a faster attentional state related to WM encoding.

### 4.3 Limitations of the study

Our results open further questions regarding the mechanisms involved in the self-initiated stimuli effect, suggesting the involvement of motor control systems and temporal prediction mechanisms. Nevertheless, our current task design cannot disentangle the contribution of each process. Our study suggests that fine temporal coupling between the motor act and the relevant stimuli triggered has a greater impact on WM processing than timely decoupled motor-sensory consequences. Nevertheless, it remains unclear what is the minimal amount of time-decoupling needed to the effect to remain maximum. In the somatosensory domain, just 100 ms motor-sensory decoupling are sufficient for modifying perception (Blakemore et al., 1999). Future research should explore the time-course of the motor-sensory consequence in self-initiation of the stimuli onset contexts.

Moreover, attention fluctuates in time (e.g., see Fiebelkorn and Kastner, 2019) or improves with increasing pre-stimuli times due to hazard function (van Ede et al., 2012). Thus, an important unexplored point in the current design is if the effect is modulated differently in even closer and further pre-stimuli onset times.

Therefore, future investigations need to be carried out to tear apart the contribution of both motor component and temporal predictability in contexts of stimulus self-initiation.

### 4.4 Neural model

Based on the results of this study, we argue that the neural mechanisms underlying the time-coupled self-initiation effect on WM might be based on motor cortex modulation on perceptual and attentional neural systems. While perceptual acceleration is related to self-initiated stimuli processing, the attentional acceleration correlates to the WM accuracy improvement seen on self-initiated stimuli. This suggests a motor modulation that provides temporal cues about the stimuli onset, which modulates both perception and attention.

Motor cortices, such as M2 in rats or the premotor cortex in primates, are anatomically connected to the visual cortex (Attinger et al., 2017; Leinweber et al., 2017). Some theories propose that motor-sensory cortex projections may act as efference copies used by an internal forward model to predict the sensory consequence of the motor act (Miall and Wolpert, 1996; Wolpert and Kawato, 1998). A proposed role for these projections is the sense of agency, allowing the nervous system to discriminate when a sensory consequence is externally or self-generated (Poletti et al., 2017). Another role for motor-sensory cortex projections is to generate time predictions about the onset of sensory consequences, acting as a marker about when a sensory change will happen (Arnal and Giraud, 2012; Saleh et al., 2010). This would possibly occur through the modulation of local field potentials in the sensory cortex, as shown in the visual cortex (Ito et al., 2011). In our data, both P1 and N1 lower latencies in time-coupled self-initiated conditions reflex early visual processing facilitation, consistent with previous data (Schroeder et al., 2010). This temporal modulation could be due to motor cortices activating motor-sensory cortex projections. This hypothesis should be tested in further studies, including methods for localizing the source of the electroencephalographic activity (which requires a greater number of recording channels) or magnetoencephalography.

Likewise, P2 and P3 earlier latencies in coupled self-initiated conditions can reflect the attentional processing's facilitation during WM encoding originated from the same motor cortices signaling. Our results suggest that this mechanism could operate in non-sensory cortex related to later attentional processing important to WM such as the dorsolateral prefrontal cortex (DLPFC) (Blumenfeld and Ranganath, 2006) and posterior parietal cortex (PPC) (Berryhill and Olson, 2008; Curtis, 2006; Todd and Marois, 2005). Motor cortices project to both DLPFC (Hasan et al., 2013) and PPC (Reep et al., 1994; Wilber et al., 2015), probably via the superior longitudinal fascicle connecting the supplementary motor cortex with the abovementioned cortices (Bozkurt et al., 2017). Therefore, movement-related activation of SMA might be signaling the onset of a relevant stimulus to be maintained to DLPFC and PPC cortices as well. Consequently, this signal may facilitate the activation of the frontoparietal network underlying attention, such that this network might have a faster activation when it receives a motor signaling. Since cortico-cortical activations through direct anatomic projections occur within a few milliseconds (Leinweber et al., 2017), this effect should be visible shortly after the activation of the motor cortex. Hence, the temporality between the movement and the relevant stimuli presentation seems to be a relevant feature of the modulatory mechanism of the active self-initiation of the stimuli, as supported by previous studies (Blakemore et al., 1999; Concha-Miranda et al., 2019; Morillon et al., 2014) and our results.

Remarkably, the motor system can modulate cognition not only using direct corticocortical connections but also through modulatory effects of motor activation on dopamine, acetylcholine, and noradrenergic circuits. The dopamine system's importance in WM functioning has been extensively documented (Arnsten et al., 2015; Brozoski et al., 1979; Sawaguchi and Goldman-Rakic, 1991; Sawaguchi, 2001). For instance, Castner and Goldman-Rakic (2004) reported a performance enhancement of WM in aged primates when administered with a D1 dopamine agonist sensitizing regime. Since the dopamine agonist was delivered by an intramuscular injection, this effect is possibly achieved by a modulation of DLPFC through the mesocortical pathway. On the other hand, dopamine depletion in monkeys' DLPFC induces deficits in visual WM tasks (Brozoski et al., 1979). Strikingly, the same authors demonstrate the reversion of the deficits when monkeys were administered with dopaminergic agonists such as L-dopa and apomorphine. Alongside this, it is known that the ventral tegmental area (VTA, which is related to the mesocortical circuit) is a signal target of motor cortices (Beier et al., 2015). Therefore, a voluntary movement could modulate dopaminergic activity, modulating DLPFC activity during active self-initiated WM encoding. Likewise, cholinergic neurons located in the basal prosencephalon show fast activity related to the current movement (Eggermann et al., 2014; Nelson and Mooney, 2016; Pinto et al., 2013). In turn, basal prosencephalon cholinergic neurons projects to DLPFC (Gielow and Zaborszky, 2017) and medial prefrontal cortex (Bloem et al., 2014) as well as to sensory cortices (Pinto et al., 2013). According to this view, a motor action could modulate movement-responsive basal prosencephalon cholinergic neurons, which in turn may modulate prefrontal and sensory cortices during active WM encoding. Finally, evidence shows locus coeruleus adrenergic neurons respond to voluntary movements (Kalwani et al., 2014; Bouret and Richmond, 2015). The adrenergic system has been correlated to enhancing long-term memory encoding (Cahill et al., 1994; Yebra et al., 2019). Since long-term memory is postulated as an important component of WM (Cowan, 2005), it is possible that it could also participate in active self-initiated WM encoding. It is also known that basal ganglia seem to participate in sensorimotor, associative, and limbic information (Jahanshahi et al., 2015; Leisman and Melillo, 2013). As part of the associative circuit functioning, basal ganglia seem to have a supporting role in WM (Constantinidis and Procyk, 2004; Marvel et al., 2019). Previous studies report projections between the associative circuit of basal ganglia and the prefrontal cortex (Dum and Strick, 2009), specifically between DLPFC and internal globus pallidus-substantia nigra pars reticulata. Although basal ganglia are organized in three different topographically separated circuits, these circuits also overlap, allowing for the integration of associative, sensorimotor, and limbic signaling (Draganski et al., 2008). Thus, the activation of sensorimotor basal ganglia circuits due to a voluntary movement could influence associative basal ganglia circuits and possibly modulate WM.

Critically, the networks mentioned above require several synaptic relays to take place and thus could unravel more slowly than the modulatory effects of direct motor-sensory/associative cortices projections. Accordingly, the catecholamine system's recruitment and basal ganglia circuits by motor systems during active self-initiated encoding could explain the discrepancy between decoupled self-initiated encoding and passive encoding in both behavior and electrophysiology. On the other hand, fast direct cortico-cortical projections can explain the distinction between time-coupled and decoupled self-initiated encoding. While stimuli appearing coupled to the movement would be modulated by both fast (direct motor projections) and slow (activity related to catecholamines and basal ganglia circuits) modulatory networks, stimuli presented hundreds of milliseconds after the movement has been executed would be influenced by the slow response modulatory networks only. This would explain that both coupled and decoupled self-presentation lead to improved WM performance but that the effect is stronger in the coupled condition.

## 5 Conclusion

To conclude, the current findings show that self-initiated stimuli effect modulates WM encoding processing through the acceleration of early sensory and attentional processes. Performance enhancement in coupled self-initiation of the stimuli onset is related to an earlier attentional state, which seems to be absent in passive states and active decoupled self-initiated states. Nevertheless, processing facilitation of early sensory and attentional processes is also present in decoupled self-initiation compared to the stimuli's passive triggering. This suggests that active self-initiation, regardless of whether it is time-locked or not, engages a motor-mediated modulation on cognition in addition to the temporal predictive mechanism (the latter being absent in the decoupled self-initiated condition). Finally, our study also remarks that sensory and attentional processing during encoding are crucial components of WM.

## Declarations

### Author contribution statement

Rocio Loyola-Navarro, Pedro E. Maldonado: Conceived and designed the experiments; Performed the experiments; Analyzed and interpreted the data; Contributed reagents, materials, analysis tools or data; Wrote the paper.

Cristóbal Moënne-Loccoz, Rodrigo C. Vergara: Analyzed and interpreted the data; Wrote the paper.

Alexandre Hyafil: Analyzed and interpreted the data; Contributed reagents, materials, analysis tools or data; Wrote the paper.

Francisco Aboitiz: Conceived and designed the experiments; Wrote the paper.

### Funding statement

Rocio Loyola-Navarro was supported by Beca Doctorado Nacional [21150153], Agencia Nacional de Investigación y Desarrollo. Support from ANID/ PIA/ Basal Funds for Centers of Excellence [FB0003] is gratefully acknowledged.

Pedro E. Maldonado was supported by Iniciativa Científica Milenio, ICM [ICN09_015], Agencia Nacional de Investigación y Desarrollo [FB210017], and BNI - ACE [210007].

### Data availability statement

Data will be made available on request.

### Declaration of interest’s statement

The authors declare no conflict of interest.

### Additional information

No additional information is available for this paper.

## Appendix A Supplementary data

Loyola-Navarro Etal 2022 Supp_V2 Loyola-Navarro Etal 2022 Supp_V2
