# Supplementary material for: Voluntary self-initiation of the stimuli onset improves working memory and accelerates visual and attentional processing
Source: Heliyon. 2022 Dec 10;8(12):e12215. doi: 10.1016/j.heliyon.2022.e12215 (PMC9791366; doi:10.1016/j.heliyon.2022.e12215)
Supplement: Loyola-Navarro Etal 2022 Supp_V2 [file mmc1.docx]

**SUPPLEMENTARY MATERIAL**

**T****able S1. Shapiro-Wilk and Levene tests p-values for each dependent variable.**

|  |  |  | **Shapiro-Wilk**  **test** | **Levene**  **test** |  |
| --- | --- | --- | --- | --- | --- |
| **Behavioral results** |  | **RT (ms)** | **p = 0.27** | **p = 0.66** |  |
|  |  | **Accuracy** | **p = 0.83** | **p = 0.59** |  |
| **ERP results** | **Amplitude**  **(μV)** | **P1-N1** | **p = 0.01** | **p = 0.9** |  |
|  |  | **P2** | **p = 2e-04** | **p = 0.52** |  |
|  |  | **P3** | **p = 2.1e-04** | **p = 0.046** |  |
|  | **Latency (ms)** | **P1** | **p = 0.05** | **p = 0.96** |  |
|  |  | **N1** | **p = 0.7** | **p = 0.58** |  |
|  |  | **P2** | **p = 0.45** | **p = 0.76** |  |
|  |  | **P3** | **p = 0.02** | **p = 0.92** |  |

**RT: reaction times.**

**Table S2.** Comparison according to having reported confidence ratings or not.

|  | **Participants with reported confidence ratings**  **(n = 15)** | **Participants without reported confidence ratings**  **(n = 10)** |  |  |
| --- | --- | --- | --- | --- |
|  | **Median**  **[C.I]** | **Median**  **[C.I]** | **t test p-value** |  |
| **Accuracy** | 0.79  [0.75 - 0.83] | 0.793  [0.77 - 0.81] | p = 0.787 |  |
| **RT** | 991.5  [937.4 - 1118.6] | 993.5  [913.4 - 1101.3] | p = 0.759 |  |
| **MoCA** | 29  [28.4 - 29.5] | 28.5  [27.7 - 29.4] | p = 0.525 |  |
| **Age** | 22  [20.3 - 23.7] | 23.5  [22 - 26.4] | p = 0.131 |  |

**RT: reaction times; MoCA: Montreal Cognitive Assessment.**

**Table S3.** Generalized Mixed Models and AIC values in Lme4 syntax

| **Models** | | | | **AIC** | |
| --- | --- | --- | --- | --- | --- |
| **Dependent Variable** | **Fixed Effects** | **Random Effects**  **(Slope\|Intercept)** |  | |  |
| Accuracy | Task + UPT * Task + BP * TL + TL * Task | (1\|SS) | 7535.6 | |  |
| Accuracy | Task + UPT + BP * TL + TL * Task | (1\|SS) | 7537.2 | |  |
| Accuracy | Task + TPT * Task + BP * TL + TL * Task | (1\|SS) | 7542.8 | |  |
| Accuracy | Task + TPT + BP * TL + TL * Task | (1\|SS) | 7544.7 | |  |
| Accuracy | Task + UPT + BP * TL + TL * Task | (Task \| SS) | 7540.1 | |  |
| Accuracy | Task + TPT * TL + BP * TL + TL * Task | (Task \| SS) | 7546.7 | |  |

Acc = accuracy; Task = encoding conditions; UPT=uncontrolled pre-stimuli time; BP= block position; TL= task learning; SS= subjects; TPT = total pre-stimuli time; * = indicates interaction between variables; AIC = Akaike information criterion.


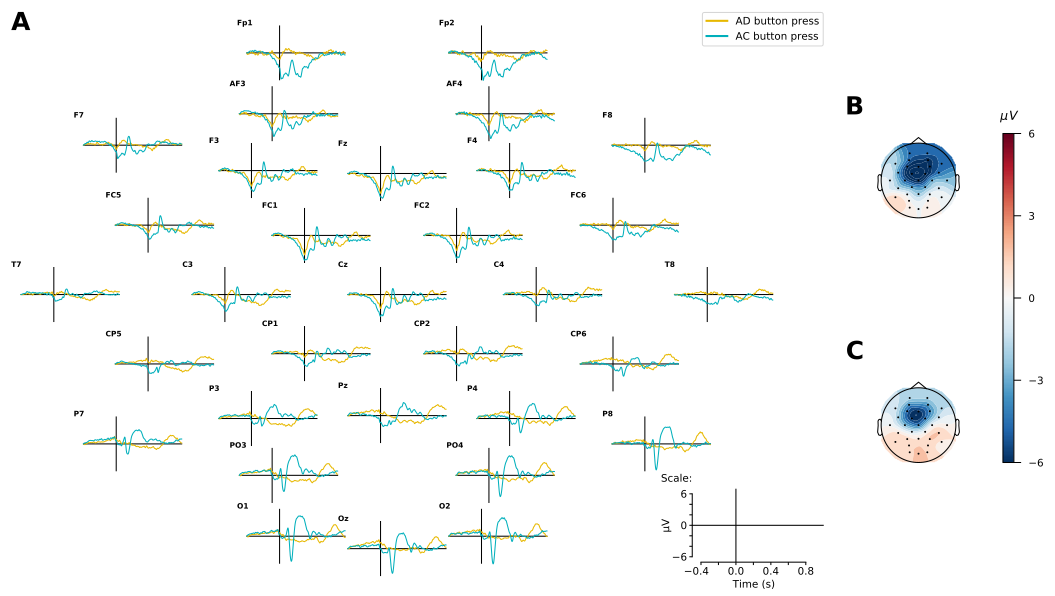


**Figure S1. Grand averages evoked by button press (t = 0 ms).** (**A**). Grand averages ERPs evoked by button press are depicted for all 32 channels. AC is depicted in light blue line, and AD is depicted in yellow line (n = 24 subjects). (**B**). Topographical plots at t = 0 ms (button press) for AC condition. red: positive voltage; blue: negative voltage; values in μV. (**C**). Same as B, but for AD condition.
